# Veganism, Moral Motivation and False Consciousness

**DOI:** 10.1007/s10806-021-09857-0

**Published:** 2021-04-19

**Authors:** Susana Pickett

**Affiliations:** School of History, Politics and International Relations, University of Leicester, Leicester, UK

**Keywords:** Veganism, Moral motivation, Akrasia, Political false consciousness

## Abstract

Despite the strength of arguments for veganism in the animal rights literature, alongside environmental and other anthropocentric concerns posed by industrialised animal agriculture, veganism remains only a minority standpoint. In this paper, I explore the moral motivational problem of veganism from the perspectives of moral psychology and political false consciousness. I argue that a novel interpretation of the post-Marxist notion of political false consciousness may help to make sense of the widespread refusal to shift towards veganism. Specifically, the notion of false consciousness fills some explanatory gaps left by the moral psychological notion of *akrasia*, often understood to refer to a weakness of will. Central to my approach is the idea that animal exploitation is largely systemic and the assumption that moral motivation is inseparable from moral thinking. In this light, the primary obstacle to the adoption of veganism arises not so much from a failure to put genuine beliefs into action, but rather in a shared, distorted way of thinking about animals. Thus, common unreflective objections to veganism may be said to be manifestations of false consciousness.

## Introduction

Why does the case for veganism often fail to convince? Insofar as it does sway opinion, why then does it fail to motivate large-scale social change? Whilst moral disagreements are inevitable, the core case for veganism from the animal rights perspective – complemented as it is by environmental, social justice, and global health considerations – is robust.^1^ Considering this jointly with commonly held moral principles, one might reasonably expect the percentage of vegans to be much higher, at least in economically developed societies. On the other hand, apathy towards veganism prevails, and common objections to veganism often rest on rationalisations (Piazza 2015, p. 114). In this paper, I suggest that a failure to accept the moral status of animals as required by veganism may itself constitute a failure of moral motivation (hereinafter referred to as motivation). Central to this position is the premise that moral thinking and motivation are inseparable, and thus thinking does not necessarily precede motivation. If this is the case, then common excuses presented against veganism express failures of motivation rather than intent, by which I mean the motivation to think of animals as being recipients of moral consideration in a manner that conflicts with our social habits and received opinion.

To narrow the scope of my opening questions, I examine the motivational problem from two radically opposing perspectives; namely *akrasia* and false consciousness. *Akrasia* – often known as ‘weakness of the will’ – is a failure of practical reasoning whereby individuals act knowingly and willingly against their better judgement. This idea has already been developed by Aaltola (2016) to explain the widespread reluctance to adopt veganism. Marxian false consciousness, by contrast, is traditionally understood as the social consciousness of an exploited class. It leads individuals to act – not fully knowingly or willingly, and thus not akratically – under a dominant ideology. This ideology may run contrary to one’s best interests, but I argue that it can also taint one’s conception of the ‘greater’ good. I understand false as applying to groups of individuals beyond social class, and argue that it is false consciousness, rather than akrasia, that is more likely to be a persistent condition that dampens motivation. As such, false consciousness may have greater explanatory power than *akrasia* for the widespread refusal to shift towards veganism.

This paper is divided into three sections. First, I offer a brief overview of the motivational difficulties associated with veganism, specifically the role of willpower and typically presented rationalisations. Second, I give an overview of *akrasia* and the structure of akratic action. Furthermore, I consider social factors which impact upon our moral thinking, serving to highlight that moral thinking is not reducible to syllogistic-style reasoning. Shortcomings of the application of *akrasia* lead on to the final section on false consciousness, wherein I explore the persistency of dominant ideologies and their impact upon moral thinking and motivation.

## The Vegan Motivational Problem

Moral motivation is typically conceived as the phenomenon of being motivated to do what one judges to be the right thing to do. Naturally, moral reasons can conflict with one’s self-interest and other reasons. In the animal ethics literature, care ethicists, including Luke (1992), are critical of the mainstream, rationalist approach exemplified by Singer (2015) and Regan (2004). The rationalist approach tends to put forward arguments for veganism and vegetarianism without tackling the motivational question of why some people may be convinced by their arguments but fail to put their beliefs into action. By contrast, care ethicists consider humans to have an innate sense of empathy towards animals, which is the basis of moral motivation, but such empathy needs to be cultivated. A problem with this approach is that most people carry on eating animals despite being empathetic to their suffering. Indeed, it is not unusual for carnivores to feel guilt and avoid imagining a slaughtered cow when eating a hamburger (Greenebaum 2012, p. 316). Hence, it is pertinent to ask why veganism poses such motivational difficulties, considering that the public possesses some moral regard for animals as well as varying degrees of empathy for animals.

## *Bona Fide* Challenges

While some aspects of veganism, such as health and environmental considerations, may be motivated by human self-interest, other dimensions conflict not only with narrow self-interest but also with prudential self-interest. As such, they constitute *bona fide* reasons to act or side against veganism. ‘Go vegan’ approaches present veganism as being easy, yet some challenges merit attention. These include financial sacrifice, social alienation, and conflict. However, I argue that taste (flavour) is not a *bona fide* reason.

First, veganism may sometimes involve financial sacrifice. This is because vegan substitutes often cost more (Mills 2019, p. 17). However, this does not apply to a large part of the population who has access to and can afford plant-based foods. Second, veganism involves alienation. Food is communal in family and social situations, and a vegan at the table can be seen as a threat (Twine 2014, p. 632). Worse still, vegans often experience exclusion and disapproval (Bresnahan et al. 2016, p. 13) and such forms of discrimination as ‘vegaphobia’ can arise (Horta 2018, p. 359). Third, veganism involves moral conflict, not only because of how vegans are perceived but also because of how they perceive others. Raimond Gaita states that vegans who provocatively shout, ‘meat is murder’ exhibit a pathological gap between what they profess and how they act, in that ‘they don’t act as though they live among murderers’ (Gaita 2016, pp. 22–23). This insight is powerful, even when applied to less polarising claims such as ‘meat involves unnecessary suffering’. From the perspective of some vegans, it can be soul-draining to inhabit a world that celebrates animal consumption and forces ‘question upon question from non-vegan interlocutors’ (Reid 2017, p. 39), and vegans are often asked to justify their standpoint and then subsequently criticised for being ‘preachy’ (Cole and Morgan 2011, p. 149). Fourth, radical factions can create tension with other individuals who do not live up to the expectations of the ‘hegemonic vegan frame’, a phrase coined by Wrenn (2019) to describe highly bureaucratised veganism (often referred to as the ‘vegan police’). There are indeed many ‘veganisms’ (Jones 2016, p. 24). Hence, vegans may face opposition, not only from non-vegans but also from other vegans.

Finally, Kazez (2018) argues that food taste is not necessarily trivial. For example, persistently unpalatable food could affect one’s wellbeing. However, I disagree that this constitutes a *bona fide* argument against veganism, because it is based on a hypothetical consideration that assumes too much since not all vegan food tastes disgusting to most people. As Singer notes, it is not as if animal flesh is uniformly delicious and vegetarian food is uniformly awful (Singer 1980, p. 333). Given this logic, one can reasonably object on the basis that taste is typically trivial when compared with what Rowlands (2013, p. 6) refers to as an animal’s ‘vital interests’. What is one to make, then, of those seemingly incapable of going vegan owing to their craving for meat? For instance, Eugene Mills recounts how he gave up after trying to be vegan for three days. His cravings for hamburgers became so powerful that he became distracted from the pursuit of important projects (Mills 2019, p. 19). It is not clear, though, that he deemed veganism to be an important long-term project.

Excepting taste, the aforementioned challenges can constitute *bona fide, prima facie* reasons for not embracing veganism. When coupled with the realisation that one’s lifestyle choices may have little positive impact globally (this is the phenomenon of ‘causal inefficacy’ which I discuss in more detail later), and after considering the disconnect between consumption, production, and killing, these reasons can become powerful. As a result, it may require substantial willpower to become a vegan against one’s cultural traditions. There are cases, however, where veganism does not require willpower. For example, where veganism is second nature (Lumsden 2017, p. 221); or one finds joy rather than sacrifice in veganism (Aaltola 2015, p. 42). In general, though, the act of becoming a vegan does require some degree of willpower.

## Willpower in Deliberation

One may object on the grounds that, if animals have no moral status, as Hsiao (2015, p. 284) proposes, then the moral motivational question of veganism does not arise. However, I disagree that this is necessarily the case. It appears to me that moral thinking and motivation are inseparable in the same way that reason and feeling cannot be fully separated, any more than form and content can. Indeed, without motivation, moral thinking would not be possible, for what else would motivate the thinking insofar as moral thinking is not purely theoretical? Hence, when I speak about moral motivation, albeit broadly conceived, I also include the motivation to deliberate about moral matters, including those concerning animals. According to this view, which I refer to as the ‘motivational unity thesis’, motivation is not always something that takes place at the end of a practical deliberation as to whether it is right or wrong to act (this is the narrow conception of motivation). Motivation is also needed to see certain *others* as worthy of moral deliberation in the first place.

The idea that animals have no moral worth is not commonplace, but the notion that animals are of lesser worth is central to the orthodoxy of animal welfare, a commonly held view which justifies animal suffering according to their utility to humans. This view has been said to explain ‘some of the apparently schizophrenic attitudes to animals that occur in Britain and elsewhere’ (Garner 2013, p. 80). Regardless of whether one believes that animals are of lesser, or indeed no moral worth (or whether one has ever considered any of this in terms of moral worth), the motivation to think things through with moral seriousness fails when we conclude that we have a right to eat or kill an animal merely because, for example, it is traditional, natural, or simply because the animal was raised on a local farm or one with higher welfare standards than some other farms.

More elaborate justifications against veganism can be provided, but we fail to do justice to animals as the objects of our deliberation if we conclude that safeguarding our lifestyle habits is generally a good enough reason to justify animal exploitation. This constitutes a broad motivational failure insofar as we fail to view animals as individuals who are ‘equally real’, to borrow Thomas Nagel’s phrase (Nagel 1970, p. 14). Still, one might lodge at least two objections. First, there is no motivational failure if it is not deemed morally objectionable to use animals as commodities in industrialised societies. Second, one might concede that a motivational failure only exists if one holds the conviction that veganism is morally obligatory, yet otherwise fails (akratically) to act accordingly.

Since this paper is not an argument for veganism, I cannot respond to the first objection directly but can link it to the second objection. To clarify, I can invoke the motivational unity thesis to argue that motivational failures can take place at the level of thinking alone (including what kind of beings to include in these considerations), and not merely when it comes to putting beliefs into action. Based on this premise, the exclusion of animals from serious moral consideration is tantamount to moral nihilism and leads only to further rationalisations when probed. Therefore, in addition to the prudential (*bona fide*) reasons against veganism discussed earlier, I now turn my attention to some common rationalisations.

## Two Rationalisations

Rationalisations against veganism readily occur when the issue is not thought through. Indeed, we are prone to motivated ignorance (Tam 2019, p. 6). The objection that animals only exist to be eaten and various other defensive tactics, exhibit apathy in the face of superior evidence to the contrary. Poor argumentation is relevant to motivation because thinking requires effort, while social habits and contempt inhibit it. Many rationalisations against veganism are merely strawmen, yet more sophisticated objections permeate the animal ethics literature, namely the causal inefficacy objection and the principle of unnecessary harm. On the one hand, causal inefficacy is the idea that an individual’s veganism has no impact on the market, specifically that one’s veganism will not make a difference to overall meat consumption. On the other hand, unnecessary harm is the principle (in the current context) by which it is unjustifiable to harm animals when vegan alternatives are available—a principle that is subject to distortion. Both principles are nonetheless interesting as they serve as a double-edged sword, both for and against veganism.

The causal inefficacy objection to veganism has accrued a vast literature which has been recently summarised by Fischer (2020). It is related to the ‘free-rider’ problem of rational choice theory, although my concern here is with the role of motivation in our thinking about causal inefficacy serving effectively as a proverbial ‘get out of jail free card’. There is a parallel with global warming, whereby people manage feelings of hopelessness with expressions such as ‘what can one person do?’, often to avoid thinking about a challenging issue (Cole & Morgan 2011, p. 156). In fact, from the existence of a global problem alone, nothing clearly and directly follows with regards to individual responsibility.

In this context, group identity can be powerful, since a group can be more impactful and offer moral support: ‘within the safe bubble of the vegan community, its practitioners are noticeably joyous’ (Twine 2014, p. 637). Relatedly, hope plays an important role in moral thinking. Moody-Adams (2017, p. 155–6) discusses the motivating power of hope, specifically how those social movements which deepened our understanding of justice and compassion were driven by those who were confident in acting on their moral convictions and hopeful of moral change. Similarly, Agnes Tam emphasises the power of “We-reasoning” as a distinctive form of communitarian rationality (Tam 2019, p. 3). Naturally, this does not mean that one abandons self-critical thinking, but it is a potential pitfall of identity groups (Fukuyama 2018, p. 115).

As Garner points out, the phrase ‘unnecessary harm’ is somewhat vague, a catch-all that can have political advantages in supporting a spectrum of speciesist positions depending on geographical and historical factors (Garner 2013, p. 81). For example, animal harm is viewed as a necessary evil in support of traditional forms of hospitality and economic interests. Central to the manipulation of these principles is the conflation of difficult, often potentially intractable empirical and analytic problems with practical moral matters about how one should live. In this vein, Reid has pointed out that simply not having a fully worked out theory of veganism is not sufficient reason, in of itself, for not becoming a vegan, in the same way as not having a fully worked out theory of knowledge is not a justification for epistemic scepticism (Reid 2017, p. 38). Indeed, veganism can be seen as a practical stance in response to animal exploitation, even though it can only ever be aspirational, for it is not possible to avoid causing harm altogether (Gruen and Jones 2016, p. 157–158). In order to reach the vegan *practical* conclusion, one need not have to resolve intractable problems of causation, collective responsibility, or necessity.

I have argued, because moral thinking and motivation are not entirely separable, that distorted thinking can dampen motivation, while motivational failures may also result in morally distorted thinking. Take, for instance, the conflation of difficult empirical and philosophical matters with practical moral considerations. Next, I consider how philosophers have traditionally accounted for the breakdown of moral motivation in practical deliberation, and how this can be applied to the vegan motivational problem.

## Omnivore’s Akrasia

*Akrasia*, sometimes referred to as a weakness of will or incontinence, is often understood to mean an intentional action contrary to one’s better judgement. It is, by definition, rather a failure of practical rationality in the shape of a motivational failure. The literature on *akrasia* dates back to ancient Greek philosophy and the contemporary literature in moral psychology is often technical. To be concise, I assume that *akrasia* is possible and follow Davidson’s (1980) definition of *akrasia* as an action that is free, intentional, and contrary to a full-blown practical judgement.

In doing *x* an agent acts incontinently if and only if: (a) the agent does *x* intentionally; (b) the agent believes there is an alternative action *y* open to him; and (c) the agent judges that, all things considered, it would be better to do *y* than to do *x*. (Davidson 1980, p. 22)

Practical reasoning often starts with *prima facie* judgements, whereupon various reasons are weighted against each other until an evaluative conclusion is derived. When deliberating whether one ought to become a vegan, *prima facie* reasons might include animal welfare, health, or environmental concerns (notwithstanding myriad other reasons for and against veganism, including one’s psychological and social wellbeing, or how one’s actions will be perceived by others). An individual may accept good overall reasons for adopting veganism, yet fail to embrace it in practice. Indeed, this seems quite plausible. Elisa Aaltola (2015) coined the term ‘omnivore’s *akrasia*’ to refer to the state arising in those who voluntarily consume animal products despite believing that they have been produced by immoral means. Could widespread *akrasia*, then, play a major role in preventing a significant proportion of the public from adopting veganism? I argue that, despite its explanatory power, the traditional approach is subject to two limitations.

## The Limits of Traditional Akrasia

A limitation of *akrasia* is that moral decisions, such as the decision to go vegan, may not necessarily be the outcome of practical deliberation. On the flip side, one’s better judgement may be faulty. In explanation, ‘all things considered’, or *prima facie* judgements may not necessarily yield a correct moral answer, not least because we are limited as epistemic and moral beings. Some philosophers (Arpaly 2000; Audi 1990; McIntyre 2006) have even questioned whether *akrasia* is necessarily irrational. What if the better judgement itself is faulty, or if the desires which ground the ‘better judgement’ fail to represent the agent’s overall desires and interests?

I shall illustrate this with a powerful example from Bennett's reflections on *Huckleberry Finn* (Bennett 1974), so that I can then explore how this applies to veganism. In Mark Twain’s famous novel, Huck believes that, all things considered, the right thing to do is to turn his slave friend Jim in to the authorities, but he fails to do so. ‘Huck hasn’t the strength of will to do what he sincerely thinks he ought to do’ (Bennett 1974, p. 126). He acts simply out of sympathy for Jim. This turns *akrasia* on its head, for Huck acts out of moral necessity (he cannot do otherwise), yet he acts against his better judgement.

Similarly, veganism may not necessarily be the direct outcome of practical deliberation. For some, the commitment to veganism may happen over and above any *prima facie* considerations. It may be the case that one already has an inner necessity. For example, one is moved by the visceral repugnance of the slaughter and ingestion of animals or a deep sense of compassion.

Thus, one could argue that the *akrasia* explanation of non-veganism involves an overly simplistic, syllogistic account of moral thinking, largely ignoring the social context. Individuals are not disembodied moral agents capable of making rational decisions independently of the social contex—there is much more at stake than merely *prima facie* reasons in terms of practical deliberations about what one morally ought to do. Could a more nuanced, socially informed notion of *akrasia* serve to overcome this limitation?

## Sociopolitical Akrasia

Aaltola (2015, 2016) takes a nuanced sociopolitical approach to omnivore’s *akrasia*. Like Amelie Rorty (1997), she views *akrasia* as a social problem, in that social forces prevent veganism by placing individuals within a continual state of *akrasia* wherein conscious deliberation and self-control are futile. These forces include ambiguity or conflict at the root of our institutions, habit, consumerism, and the culture of immediate reward or sensory hedonism. Significantly, the meat-eaters’ paradox, in which a societal love for certain animals such as dogs and cats is cultivated, while cows, pigs, and other animals, which are equally sentient, are mistreated and slaughtered, is entrenched within our institutions (Aaltola 2016, p. 118).

Despite these conflictual beliefs,^2^ most individuals believe that food choices are rational but overlook how these choices are grounded via emotive, cultural, or otherwise more ambiguous justifications (Aaltola 2016, p. 117). Habit perpetuates the meat-eaters’ paradox for, although the original reason for eating meat was survival, it is no longer essential for a large part of the world’s population, so it is in some ways a mindless habit and one that is exacerbated by consumerism. Given this, asking individuals to exercise self-control is insufficient (Aaltola 2016, p. 124). Indeed, ‘our akratic choices may take place beyond the possibility of conscious deliberation, and thereby beyond the possibility of conscious hedonism or egoism’ (Aaltola 2016, p. 131). This results in a vicious circle wherein contempt may feed moral apathy and we may thus become apathetic to act altruistically. Therefore, Aaltola (2016, p. 135) concludes that we are in a state of continual *akrasia*.

Whilst such application of *akrasia* is insightful, *akrasia* may not be the best explanation for the phenomenon of widespread omnivorism. Crucially, the possibility of perpetual *akrasia* seems absurd, especially given that *akrasia* is, by definition, free intentional action contrary to one’s better judgement. In the context of permanent *akrasia*, as described by Aaltola, individuals are not acting freely or intentionally, and their better judgement is not to become vegans. As such, they are not *akratically* failing to become vegans: they never set out to do so in the first place, so there is no motivational failure as the rational outcome of practical deliberation.

Similarly, *akrasia* may not be the best notion to incorporate mindlessness, self-deception or voluntary ignorance. The notion of *akrasia* struggles to accommodate the fact that not all our thinking is transparent, *bona fide*, or easily moulded into practical syllogisms. For instance, it has been said that, once we are accustomed to behaving in ways that have implicit normative content, we struggle to contemplate the possibility of change and may thus engage in self-deception to justify wrongful actions (Cooke 2017, p. 9). John Searle exemplified one such deception: ‘I try not to think about animal rights because I fear I’d have to become a vegetarian if I worked it out consistently.’ (Cooke 2017, p. 10).

Indeed, such deception is more likely to be widely shared, given that most people give similar excuses against veganism, commonly referred to as the 4Ns (the belief that eating meat is natural, normal, necessary, and nice; Piazza et al. 2015). For Luke (1992, p. 106), such rationalisations consume abundant social energy. However, one can object that very little thinking power is normally used, even though the passions may be inflamed. Given these limitations, one must ask whether the notion of false consciousness would fare any better in accounting for such persistent motivational gaps and largely unreflective responses to veganism or be more cohesive with the idea that animal exploitation is largely systemic.

## Omnivore’s False Consciousness

False consciousness is a post-Marxian notion. Although Marx did not use the phrase ‘fase consciousness’, the notion is embedded in much of his thinking. Thus, Miller (1972, p. 433) argues that a broad interpretation of the related concept of ideology, understood as applying to theories, belief-systems and practices involving the use of ideas, has great explanatory power concerning the persistency and influence of ideologies over the actions of the groups who adopt such ideologies. Crucially, if such a group is confronted by others holding incompatible ideas, ‘it has no resources to fall back upon, it can only reaffirm its original faith’ (Miller 1972, p. 433). Alternatively, if the ideology is seen primarily as an explanatory framework, then ‘the ideology is given repeated empirical confirmation, through the selection of what is perceived’ (Miller 1972, p. 433). When ideologies function in these ways, they can be said to involve false consciousness. If Miller is correct, and omnivorism can be shown to depend on an ideology that necessarily involves false consciousness, then this may account for the persistency of omnivorism over reasoned arguments, thus filling the gaps left by omnivore’s *akrasia*.

In Marxist theory, false consciousness is essentially deemed to be political in nature and refers to the social consciousness of the proletariat as an exploited class under capitalism. It is thereby related to the concept of ideological power and forms the basis of Luke’s third dimension of power, wherein the illegitimate use of power by one group over another confers the power to mislead (Lukes 2005, p. 149). To put it simply, it is the power to control what groups think as being right, resulting in biased acceptance without question. Marx and Engels used the concept of ideology to refer to ‘the distorted beliefs intellectuals [hold] about society and the power of their own ideas. Those who produced ideologies suffered from false consciousness: they were deluded about their own beliefs.’ (Eyerman 1981, p. 43). Given this tenet, one may be puzzled by my use of false consciousness, as it seems to shift the construct of veganism to being about people rather than about animals. How, then, is false consciousness relevant to the problem of motivation in veganism, given that animals are the exploited group in question, even to the extent that some theorists, such as Perlo (2002, p.306), have likened animals to the proletariat?

The notion of false consciousness has evolved since its origins, and my intention here is to expand its application further. Marx’s concept was further developed by Gramsci, Lukacs and the early Frankfurt School, and later expanded to apply to any social class with a ‘limited form of experience in society’ (Eyerman 1981, p. 43–44). Thus, it is not limited to Marxian class and has been more applied broadly to groups both before and after the rise of capitalism. For example, Michael Rosen (2016, p. 10) sees Marxian false consciousness as a critique and the development of rationalistic understandings of a previously unformulated notion of false consciousness, beginning with Plato, for whom irrationality of the soul led to the injustices of the state; and Aristotle, for whom false consciousness is necessarily *akratic*. Omnivore’s false consciousness may thus be viewed as a novel development and a particular application of false consciousness^3^ to a broad majority of humans who practise omnivorism in economically developed societies.

## Narrow and Broad False Consciousness

So, what then is false about false consciousness? False consciousness is often portrayed in terms of one being misled about one’s true interests. However, there is a distinction arising between being blinded by one’s interests (i.e., being impetuous) and being blind to them, where false consciousness is often associated with the latter (Runciman 1969, p. 303). The self-interest interpretation, however, omits the altruistic and moral dimensions of human thinking, whereby one may also be blind not only to others’ interests but also to their moral dimension. Traditionally, false consciousness is about group interest and social ontology, but I shall argue that it can also distort moral thinking in much the same way as it distorts non-moral thinking. The notion that Marxism is not totally abstracted from morality is not novel (e.g., Lukes 1985), so I will instead set the context before I explain how it bears on veganism.

Marx avoided talk about morality, not only because he hated preaching and was distrustful of the moralist per se (Popper 1995, p. 220), but because he saw contemporary morality as being part of the bourgeois superstructure, in which class morality added an extra layer of false consciousness. The worker believes, according to Singer, that capitalist has a moral right to the profits^4^ (Singer 2018, p. 83). Although Lenin and others claimed that Marx’s theory was purely scientific, it has since been argued that Marx held a normative position, not least because of his desire to end capitalism (Cochrane 2010, p. 95; Singer 2018, p. 82), his hatred of servility, and his ‘desire for a better world that it is hard not to see as moral’ (Lukes 1985, p. 3).

Central to the Marxian notion of false consciousness is the tenet that both the capitalist and proletariat are afflicted by it and, thus, that the proletariat believed, whether implicitly or explicitly, that the capitalist had a moral or legitimate right to profit. If proletarian Jim held such a belief about himself, he would also believe that the capitalist had a right to the labour of his fellow proletarians. In this world view, the proletariat is both wronged by the capitalist and unaware that they have been wronged. Similarly, capitalists had so distorted or delimited moral ideas insofar as they too failed to acknowledge the true interests of the exploited group and were unaware of their wrongdoing. In the case of animals, the public largely carries on supporting systemic practices of animal-exploitation without acknowledging the wrongs inflicted on animals in its name.

Hence, false consciousness may be understood narrowly as relating to either self or group interest or, more broadly, as including an altruistic moral dimension in the sense of limiting such a dimension. Indeed, if I am blind to my own true interests, then I may not necessarily be receptive to those of other people or those of animals. My claim is not that there is a causal link between blindness to one’s own interests and blindness to the interests of others, but rather that it is absurd to contend that false consciousness impacts only one’s self-interested thinking. Crucially, false consciousness may so taint one’s conception of the good and limit the moral self, that it has the effect of occluding the motivational difficulties of veganism. Hence, the *akratic* break (motivational failure) does not actually take place, at least not explicitly.

This broad interpretation of false consciousness presupposes a close link between alienation and false consciousness. As Rosen states in his discussion of Marx’s early writings on alienation as a form of life, ‘the alienated worker’s failure to recognize himself in the product of his labour and the failure of isolated individuals to recognize each other fully as fellow human beings are expressions of false consciousness that are lived and experienced before they are theorized about or reflected upon.’ (Rosen 2016, p. 35). In this sense, the moral self is not impervious to false consciousness. This is interesting within the context of the vegan debate, as the cumulative case for veganism (i.e., the case from a wide range of perspectives) encompasses both moral and enlightened self-interested strands. If we deem both the narrow and broad sense of false consciousness to be appropriate, then this may help to explain how a substantial proportion of the general public may be somewhat blinded by the dominant animal-exploiting ideology in contrasting, yet complementary ways, so as to render the ideology quite impenetrable.

This narrow sense of false consciousness applies to the case for veganism from either anthropocentric or enlightened self-interest perspectives. Strictly, these perspectives support plant-based living as opposed to fully blown ethical veganism but are largely consistent with it. Overall, exploitative animal practices are agreed to have a detrimental impact on the environment, sustainability, and climate change (Rosi 2017; Sabaté & Soret 2014), as well as global human health (Tuso 2013) and that of future generations (Deckers 2011). Zoonotic diseases such as severe acute respiratory syndrome (SARS) and coronavirus disease (COVID-19) have also been traced to wet markets where animals are confined within unnatural and unsanitary conditions (Singer 2020, pp. 82–83). Despite these, and other harms to humans, the animal agricultural complex has a vested interest in continued animal exploitation. Moreover, the advertising industry and media can exercise tremendous power in perpetuating the desire to consume animal products.

There are at least two difficulties with the attribution of narrow false consciousness in these scenarios. First, the oppressor and oppressed (or exploiter and exploited) groups are not distinct, for at least some humans count as the exploited, even though they too contribute to animal exploitation through their consumption and labour. Although this complicates matters, it does not in of itself make the premise of false consciousness impossible, for (unlike Marxian social class) an individual can belong to more than one group at any one time. In this respect, animals are posited as the oppressed, yet humans are both oppressor and oppressed. In fact, the presumption of such a stark dichotomy of classes would have very little application in terms of the animal agricultural complex which lacks any clearly defined boundaries.

Second, false consciousness is supposed to affect both the exploiter and exploited alike, but it is not altogether clear why it would not be in the interest of the exploiter to exploit, particularly in terms of material self-interest. It may well be that the exploiting group is subject to false consciousness but is not necessarily deceived about its own material self-interest. After all, many people’s livelihoods depend on animal agriculture, which does not go against their immediate, material self-interest. However, the exploiter might be in denial about the consequences of their own exploitation. In Hegel’s dialectic, which influenced Marx’s thinking, the master (to his own detriment) becomes too dependent on the slave. When translated in terms of the current exploitation of animals and nature, exploiters act in such a way as though they are blind to the ultimate consequences of their actions, yet the crucial difference here lies between enlightened self-interest in the medium term and the long run, for it is the latter that false consciousness is supposed to affect.

On the other hand, in a somewhat broader sense, false consciousness acts against the case for veganism from the point of view of ethical and political perspectives such as animal rights and care ethics. These are deemed to be ‘veganism for the animals’ perspectives that constitute the core of ethical veganism, which are not defensible from the standpoint of self-interest. In this context, false consciousness might serve as a good explanatory match for two phenomena; namely the absence of moral reflection on whether one ought to become a vegan (in light of the meat-eater's paradox), and second, the poverty of thinking exemplified by the public’s common rebuttals in response to arguments for veganism.

Although not all objections or negative responses to veganism are crude, there is a widespread social malaise in the form of a prevalent moral apathy towards the exploitation of animals. This matter is political, not only from the perspective that humans exercise illegitimate power over animals but also that animals are worthy of political justice as argued, for example, in *The Political Turn in Animal Ethics* (Garner and O'Sullivan 2016). Further, it could be construed that the public’s commonplace objections to veganism are socially determined and thus often devoid of individual self-expression. The issue is also a very personal one, in the sense that moral thinking is inextricably personal, yet such thinking may at times be thwarted by sociopolitical imperatives. When deliberating on whether one ought to become a vegan, insofar as one engages in moral discourse at all, the moral problem is, and ought to be, inescapably one’s own in the sense that one cannot pass it on to someone else to resolve on one’s behalf (on this topic see Gaita 1989, p. 128), let alone rely on the unexamined opinions of the majority. However, this is precisely what tends to happen when people confront veganism. The next step, then, is to relate common, unreflective objections to veganism to aspects of political false consciousness.

## Four Features of False Consciousness

To deconstruct how thinking can be systematically distorted, I build on Miller’s account of the four dimensions of false consciousness (Miller 1972, p. 443–444), sketching how these features may be manifested in omnivore’s false consciousness. The four interrelated features are conceptual inadequacy, isolation of phenomena, eternalisation, and reification.

First, false consciousness involves a degree of conceptual inadequacy in that it leads to fallacious reasoning**.** For example, generalisations based on superficial similarity, whereupon subsequent analysis can reveal them to be disparate. Conceptual inadequacy includes such common injunctions against veganism as animals being unintelligent, carnivorism natural, and vegans self-righteous. These claims expose distortion as empirical analysis – and frequently linguistic or logical analysis alone – can prove them to be fallacious.

For instance, does it follow from the premise that animals are less intelligent that we have a moral right to eat them? Does the fact that something is natural necessarily make an action or attitude morally justifiable? Are *all* vegans self-righteous? Even if they all are, this latter argument is effectively *ad hominem* and therefore invalid. Similarly, the idea that veganism is impossible because nobody can ever avoid partaking in harming animals is to misunderstand the very concept of veganism. It exhibits fallacious reasoning by misusing the concept of vagueness. Just because there are borderline cases between a child and an adult, or shades of grey, it does not necessarily follow that nobody can ever be an adult, or that nothing can be truly black. The same holds true for veganism. While nobody would seriously deny that adulthood or true blackness are possible, many are prepared to subject veganism to a *reductio ad absurdum*. These common examples of conceptual inadequacy are not isolated mistakes, or merely manifestations of the ignorance of specific information, but rather are fundamental ways in which thought fails. They are manifestations of how the acceptance of the moral and political legitimacy (or neutrality) of animal exploitation is deeply rooted within the collective consciousness and embedded within our social institutions.

Second, the process involves the isolation of phenomena, notably a refusal to see an instance of individual behaviour as being part of a wider social system. For example, the belief that one exercises free will in consumer choices^5^ and, therefore, that one’s decision to eat animals is autonomous when one is, in actuality, making socially conditioned decisions which are influenced by the meat industry. Hence, Nibert talks of a socially engineered public consciousness, highlighting how organisations such as the ‘Center for Consumer Freedom’ exploit both the concepts of ‘freedom’ and ‘consumer choice’ (Nibert 2013, p. 266). Since others are doing the same, these attitudes are considered to be justificatory of the wider system.

Third, it involves eternalisation, whereby conventional relationships or characteristics are regarded as being permanently fixed within the nature of things. For example, in medieval Europe, society was ranked hierarchically from God down to inanimate objects. Similarly, the hierarchical belief in speciesism is effectively an extension of the belief that ‘might is right’, wherein biological omnivorism is extrapolated to entail a right to exploit animals. For Cooke, the view of the innate inferiority of animals is embedded within our social consciousness, and the moral imagination must be cultivated to break out of such self-deception (Cooke 2017, p. 14–15). This feature of false consciousness serves as the key to perpetuating certain practices.

Let us consider an example of eternalisation, such as the common belief (in some countries) that a turkey must be the centrepiece of the Christmas dinner table, as tradition dictates, in such a way that a vegan alternative is deemed to be out of the question. In what way is this thinking distorted? How does it manifest as a form of false consciousness? One of the distortions revolves around the false belief that tradition is alone sufficient justification for engaging in a specific practice. Some traditions, such as forced marriages, are morally wrong and so tradition alone does not morally justify a practice. It constitutes a distorted form of thinking rather than a question of holding a false belief, as most individuals living in liberal societies do accept that tradition alone does not morally justify a practice. It manifests as a form of false consciousness insofar as the distortion is not politically neutral.

Like most animal agriculture, the mass confinement, fattening and slaughter of hundreds of millions of turkeys aged between 14 and 24 weeks for Christmas involves the illegitimate use of power of humans over animals. Yet, such traditions continue, not only because people enjoy certain flavours and family traditions, but also because a powerful industry lobby has a vested interest in perpetuating and normalising this form of animal exploitation. For example, in December 2019, the UK’s National Farmers Union (NFU) took issue with a BBC commercial in which a cartoon turkey wearing an ‘I Love Vegans’ sweater announced ‘less of us have been gobbled this year’ (The Telegraph 2019). The NFU feared that the BBC was promoting a political view. What was not questioned, however, was that the farming and killing of animals may not be a politically neutral standpoint.

Finally, it involves reification. It reduces individuals to the status of mere objects of fixed properties, their individuality denied, similar to the archetypal Nazi depiction of the Jew (Miller 1972, p. 444). Animals, too, are objectified when reduced to the status of commodities such as forms of food or modes of transportation, or even being owned as pets. As expressed by Cole and Morgan (2011, p. 149), ‘ethics are simply ruled out of order by the prior to objectification and invisibilisation of nonhuman animals that speciesist material and cultural practices instantiate’. This takes place on a large scale, even when people are generally aware that animals such as the Christmas turkey are (or rather were) individuals, not mere things. Still, animals are essentially commodified, an idea that also links into Marx’s theory of commodity fetishism.

Miller’s analysis provides a framework for dissecting how common objections to veganism, and the belief systems that ground them, are distorted and thereby largely unmovable. It gives weight to the idea that these objections manifest false consciousness. As a form of political false consciousness, omnivore’s false consciousness involves distorted and limited forms of thinking that are not often scrutinised. I have only touched on a small number of common objections to veganism, although there are many others, such as those exemplified in a defensive omnivore board.^6^ When one of these notions is challenged, many more excuses are proffered.

What these distorted forms of thinking lack in terms of sobriety they make up for in intuitive persuasiveness by conforming to a widely accepted worldview or way of life. According to this worldview, nonhuman animals are inferior to human animals in politically significant ways that accord the latter the moral entitlement to exploit the former. As Miller recognises, one cannot easily fight instances of false consciousness by pointing out isolated errors. Thus a broader stance is needed, yet it may not be possible to avoid false consciousness altogether (Miller 1972, p. 444). Therefore, one might ask what makes false consciousness not only possible but also so persistent and prevalent?

## The Persistency of Ideologies

The link between false consciousness and ideology is key to its persistency. Gauthier (1997, p.27–28) points out that the notion of an ‘ideology’ is employed inconsistently, yet is generally regarded as a pejorative aspect of our consciousness. He sees ideology as a theoretical construct, part of the ‘deep structure of self-consciousness’, that is, the capacity to conceive oneself relative to others and therefore to act in light of this conception of oneself as a member of the human species. Although it can be the subject of reflection, it is necessarily pre-reflective. This sounds puzzling, but Gauthier sees a similarity between ideology and language in that ‘both conceal a deep structure which unconsciously affects conscious activity’ (Gauthier 1997, p. 28). Even if one cannot think outside the boundaries of a specific language or ideology, reflection and critique are still possible, thereby enabling moral progress.

Like languages, ideologies also promote social commonality. One of the main functions of social institutions is to maintain and transmit a common ideology (Gauthier 1997, p. 28). Hence, individuals with very different ideologies, such as vegans and non-vegans, may find communication difficult. Moreover, for Marx, an ideology was not merely false but served an intentional role both in upholding the extant social order (Rawls 2008, p. 361) and continuing the *status quo* in terms of the exploitation of the proletariat. For example, hiding the act of robbery within the construct of capitalism is essential. Similarly, exploiters of animals do not want to be perceived to be exploitative, whether these agents be the state or the lawmakers protecting animal-exploiting institutions. Farmers’ associations have privileged access in terms of shaping the viewpoint of the media and in influencing agricultural policy and legislation (Benton 1993, p. 160). For example, both the US and Australia have introduced ‘ag-gag’ laws that essentially criminalise the dissemination of information about the treatment of animals (O'Sullivan 2016, p. 53). Moreover, the institutionalised praise of exploiters and punishment of animal liberationists is not a morally neutral position with regard to conceptions of the good that liberal states purport to do. As Schmitz says, ‘the animal question debunks the appearance of neutrality’ (Schmitz 2016, p. 42).

If we interpret ideologies as being pre-reflective, this aids in explaining their persistency and evasiveness from rational argumentation. As Miller suggests, repeated selective perception confirms the ideology (Miller 1972, p. 433), yet it is difficult to construct a simple verification or falsification test, as ideologies are false at the level of the whole (Miller 1972, p. 435). As such, they are not a mere set of commonly held ideas, but rather embody attitudes, common behaviours, and practices. Thus, the ideology that dominates our relationship with animals in developed societies gives rise to a level of false consciousness. It is pre-reflective in that societies embrace omnivorism without perceiving the moral need to justify it, although it is possible to reflect on it. When the dominant ideology is challenged, rationalisations can ensue. Since an ideology is not a specific set of beliefs that can be proven to be true or false in isolation, it is very difficult to ‘prove’ that omnivorism is morally wrong, or that veganism is right in such a way that any rational moral agent could be convinced.

One might object to the premise that attributing false consciousness is arrogant, for it requires a privileged perspective in terms of intellect and education. As Polsby states, ‘the presumption that the “real” interests of a class can be assigned to them by an analyst allows the analyst to charge “false consciousness” when the class in question disagrees with the analyst’ (Polsby 1963, p. 22–3). However, is the attribution of false consciousness necessarily arrogant? Lukes (2005, p. 149–150) argues that recognising the possibility of false consciousness is neither condescending, nor inherently illiberal, or even paternalistic. He considers, for example, J.S. Mill’s analysis of the subjection of Victorian women to the rule of men (in Mill 2009 [1869], p. 25) which can be interpreted as showing how most women were subject to false consciousness in the form of voluntary servitude, as opposed to coercive power. In light of such historic examples, and the fact that gender equality is now largely undisputed, the objection from arrogance is begs a question in that it denies the possibility that anyone might ever be politically deceived. It is *ad hominem* insofar as it attacks the character of the analyst, not the soundness of their views. Similarly, if future generations were to embrace the cause of animal rights and veganism, the attribution of an omnivore’s false consciousness to previous generations may then not seem too paternalistic.

Some Marxists could argue that the notion of false consciousness simply does not apply here. That may well be the case if indeed false consciousness is taken literally in a Marxist context. Instead, I have argued that there is a broad reading of false consciousness according to which it can narrow the moral self precisely because the interests of animals are not perceived in such a way as to trigger the moral motivation to practice veganism. In fact, I have attempted to detach the concept from Marxist theory as far as possible, so that one does not have to embrace Marxism in order to be able to accept how such a concept (and related concepts) may command useful explanatory power where the notion of *akrasia* falls short. 1

If there is such a thing as omnivore’s false consciousness, it would seem to follow that animal liberation (from human oppression) requires human liberation from omnivore’s false consciousness. Broad false consciousness may need to be confronted head-on through practices that promote more reflective and altruistic thinking (Cooke 2017). Narrow false consciousness, on the other hand, may be tackled directly by promoting some of the benefits of plant-based living (Fetissenko 2011), or indirectly by creating the conditions that normalise such a lifestyle (Lumsden 2017), for example, by making the shift from animal to plant agriculture easier and more desirable for farmers, or through the technological development of realistic alternatives to culling animals (e.g. in vitro meat; see Milburn 2016). A drawback of the self-interest approach, however, is that it only favours animals contingently in those instances where enlightened human self-interest happens to be convergent with those of animals. These challenges make a global shift to veganism not only fraught but also currently inaccessible to those on the opposite side of the debate. Considering how humans have habitually exploited animals, the future for most animals looks grim. On the other hand, social movements depend on hope and persist in the belief in moral progress has been said to be a regulative concept (Moody-Adams 2017, p. 154).

## Concluding Remarks

Starting from the assumption that there is a strong case for veganism in the literature, and the hypothesis that moral thinking and motivation are inseparable, I have considered how *akrasia* and false consciousness are ‘conceptual pathways’ through which our practical thinking about animals is distorted. Omnivore’s *akrasia* leaves some important gaps, for it is delimited to free and voluntary action against one’s better judgement. As such, the phenomenon of widespread omnivorism in developed societies may be better explained in terms of omnivore’s false consciousness (but I am not thereby suggesting that animal liberationists should embrace Marxism). Where omnivore’s false consciousness arises, there is no clear or explicit motivational failure to become a vegan, precisely because there is insufficient reflection for an *akratic* break to take occur. Further work in the field of moral psychology is evidently needed to unravel the motivational unity thesis, a theorem upon which this paper leans heavily.

Insofar as veganism expresses an ideology, it cannot be proven either to be true or morally right through arguments alone in such a way as to persuade any rational being or otherwise fully-fledged moral agent. Veganism is, as such, not an analytic truth to be derived from abstract moral principles but rather a moral way of life. Arguably, it is also a moral requirement. Principles such as causal inefficacy and unnecessary harm can be turned against veganism via analytic rationalisations which exploit scepticism and err on the side of narrow human self-interest, rather than an altruistic stance towards animals. Despite difficult technical and analytic considerations, one can experience veganism as an inescapable imperative; as a spiritual necessity; or as a powerful political identity against the oppression of animals. As such, some animal advocates may feel utter despair and therefore struggle to comprehend how others are not similarly moved. They may experience helplessness as to why common reasons against veganism are so weak. This paper is but one expression of such puzzlement, and a first attempt to make sense through the hitherto underexplored notion of false consciousness within the field of animal ethics.

## Footnotes

1 For a concise exposition of the cumulative case for veganism see Stephens (1994). For more recent arguments see Francione (2008), Huemer (2019) and Singer (2020).

2 There is no conflict if animals are viewed and treated only according to their purpose to humans, but it can be argued that this is how things are (the animal welfare orthodoxy), not how they ought to be.

3 False consciousness is often assumed without explanation in the Critical Animal Studies (CAS) literature (e.g., Nibert 2002, p. 247).

4 Marx may not have thought that the proletariat held such explicit beliefs given that they had no access to the superstructure, but the relevant idea is that the proletariat was blind to their interests.

5 Vegans too can be consumerist.

6 A compilation of poor excuses against veganism such as ‘we have carnivore teeth’. For an example see https://vegansaurus.com/post/254784826/defensive-omnivore-bingo.
